# Hybrid Discrete Wavelet Transform and Gabor Filter Banks Processing for Features Extraction from Biomedical Images

**DOI:** 10.1155/2013/104684

**Published:** 2013-04-15

**Authors:** Salim Lahmiri, Mounir Boukadoum

**Affiliations:** Department of Computer Science, University of Quebec at Montreal, 201 President-Kennedy, Local PK-4150, Montreal, QC, Canada H2X 3Y7

## Abstract

A new methodology for automatic feature extraction from biomedical images and subsequent classification is presented. The approach exploits the spatial orientation of high-frequency textural features of the processed image as determined by a two-step process. First, the two-dimensional discrete wavelet transform (DWT) is applied to obtain the HH high-frequency subband image. Then, a Gabor filter bank is applied to the latter at different frequencies and spatial orientations to obtain new Gabor-filtered image whose entropy and uniformity are computed. Finally, the obtained statistics are fed to a support vector machine (SVM) binary classifier. The approach was validated on mammograms, retina, and brain magnetic resonance (MR) images. The obtained classification accuracies show better performance in comparison to common approaches that use only the DWT or Gabor filter banks for feature extraction.

## 1. Introduction

Computer-aided diagnosis (CAD) has been the subject of a lot of research as a tool to help health professionals in medical decision making. As a result, many CAD systems integrate image processing, computer vision, and intelligent and statistical machine learning methods to aid radiologists in the interpretation of medical images and ultimately help improve diagnostic accuracy. These systems have been employed to analyze and classify various types of digitized biomedical images, including retina [1, 2], mammograms [3–5], brain magnetic resonance images [6–8], skin cancer images [9, 10], lung images [11, 12], and ulcer detection in endoscopy images [13, 14], just to name a few.

The typical CAD process starts with a segmentation stage to identify one or more regions of interest (ROI) in the image of interest. Then, the ROI(s) is processed for image enhancement and/or feature extraction before classification. Because the segmentation step requires prior knowledge of discriminant image features and its implementation typically calls for numerous parameter settings, recent works have attempted to eliminate it. These approaches realize feature space reduction by applying one or more transforms to the whole image and extracting the feature vector to classify from one or more of the obtained components [3, 5, 7–14].

Texture analysis has played an important role in the characterization of biomedical images. Texture analysis methods can be categorized as statistical, geometrical, and signal processing types [14]. Statistical methods are mainly based on the spatial distribution of pixel gray values, while geometrical approaches depend on the geometric properties of texture primitives. As for signal processing methods, they use texture filtering in the spatial or frequency domain to extract relevant features.

Multiresolution analysis is the most widely employed signal processing technique for characterizing biomedical images due to its capability to obtain high time-frequency resolutions. The wavelet-transform family methods are typical examples of multiresolution analysis techniques. The basic wavelet transform [15, 16] starts with a basis function, the mother wavelet, and decomposes a signal into components of different time and frequency scales; longer time intervals are used to obtain low-frequency information and shorter intervals are used to obtain high-frequency information.

The most commonly used wavelet transform in biomedical image processing is the discrete wavelet transform (DWT) [14] whose discrete time shifting and stretching variables lead to a sparse and efficient representation. The DWT takes an input image and decomposes into four subimage components that characterize it for different orientations in the horizontal and vertical frequency axes. The process can be repeated with one or more subimages if needed. More precisely, the DWT decomposition yields the approximation subband (LL), the horizontal detail subband (LH), the vertical detail subband (HL), and the diagonal detail subband (HH). These describe, respectively, the low-frequency components in the horizontal and vertical directions, the low-frequency components in the horizontal direction and high-frequency components in the vertical direction, the high-frequency components in the horizontal direction and low-frequency components in the vertical direction, and the high-frequency components in both directions. Thus, in essence, the standard DWT algorithm yields horizontal, vertical, and diagonal directional information about the frequency spectrum of an image. However, these three directions may not be sufficient to express all the directional information in digital images, particularly biomedical images [4, 14]. In an attempt to express the directional features more efficiently, several directional wavelet systems have been proposed. These include the Gabor wavelets [17], the dual-tree complex wavelet transform (DT-CWT) [18], the ridgelet [19], the curvelet [20], and the contourlet [21]. There exist also reports on biomedical applications of Gabor filter banks [22], DT-CWT [4], ridgelets [23], curvelets [24], and contourlets [5].

The two-dimensional (2D) Gabor filter decomposes an image into components corresponding to different scales and orientations. As a result, it captures visual properties such as spatial localization, orientation selectivity, and spatial frequency. The 2D Gabor filter has real and imaginary parts and is highly flexible in its representation as its parameters can be adapted to the structure of the patterns that one wants to analyze in the image. It is however difficult to find the optimal set of parameters to characterize a given image. In comparison, the DT-CWT transform provides directional selectivity, shift invariant features, and complex images. However, it suffers from limited orientation selectivity [25] and redundancy of information [26]. The ridgelet transform is appropriate to capture radial directional details in the frequency domain; in particular it is optimal for representing straight-line singularities. However, those structures are not dominant in medical images and are rarely observed in real world images. This limits the suitability of the ridgelet transform to characterize the texture of real images [27]. The curvelet transform is an extension of the ridgelet transform for detecting image edges and singularities along curves while analyzing images at multiple scales, locations, and desired orientations. It is particularly suitable for image features with discontinuities across straight lines. Unfortunately, the curvelet transform is highly redundant [28] and only few choices of mother functions are available for the curvelets as opposed to the many choices available for the standard wavelet transform [29]. Finally, the contourlet transform can capture directional details and smooth contours in a given image. In particular, it is suitable in the analysis of images containing textures and oscillatory patterns. Its main drawback is the high degree of information redundancy and occurrence of artefacts [30, 31].

In past works, we proposed several transform-based approaches to account for directional features in classifying biomedical images. For instance, in the case of brain magnetic resonance images, we proposed a simple methodology in [32, 33] where features are extracted from the LH and HL components of the DWT instead of the more common LL, or image approximation, component. We found that the LH and HL coefficients are efficient at characterizing changes in the biological tissue and help distinguish normal and abnormal image textures. For mammograms, we investigated in [34] a hybrid processing system that sequentially uses the discrete cosine transform (DCT) to obtain the high-frequency component of the mammogram and then applies the Radon transform (RT) to the result in order to extract its directional features. The validation results showed that the RT helps improve the recognition rate of the detection system. In subsequent work, we combined the DWT and RT transforms [35]. The approach targeted the HH component of the DWT decomposition and improved classification accuracy when compared to using the DWT or RT alone or the DCT-RT used in [34]. Our previous works clearly showed that directional information helps improve classification accuracy. In addition, the DWT-RT detection system was more efficient for classifying normal and abnormal images than the DCT-RT, possibly because of the multiresolution capability of the DWT and the fact that it leads to a sparser signal representation than the DCT. Still, the RT cannot capture spatial frequency, a potential feature to improve further the classification accuracy.

In this paper, we describe a hybrid biomedical image processing and classification system that uses both the DWT and Gabor filter as directional transforms and statistical features derived from them for the classification task which is accomplished by support vector machines (SVMs) [36]. As stated before, the DWT is powerful at providing sparse and efficient image representations [14]. However, except for the LH and HL subbands whose coefficients depend on image row and column information, respectively (an effect of the subband coding used by the algorithm), the standard DWT is essentially an image compression tool and it cannot perform directional analysis at arbitrary directions. On the other hand, the Gabor filter can process images in terms of preferred orientations at arbitrary spatial frequencies. Moreover, it provides nonredundant information and can offer high directional selectivity. Thus, combining DWT and Gabor filter banks in sequence may lead to improved feature extraction from biomedical images and better classification of normal versus abnormal images in comparison to using DWT or Gabor filter banks alone. In this hybrid processing scheme, the DWT acts both as high-frequency filter to extract abrupt changes in image texture and image compression engine to reduce image dimensionality and a Gabor filter bank extracts the directional information.

**Figure 1:**
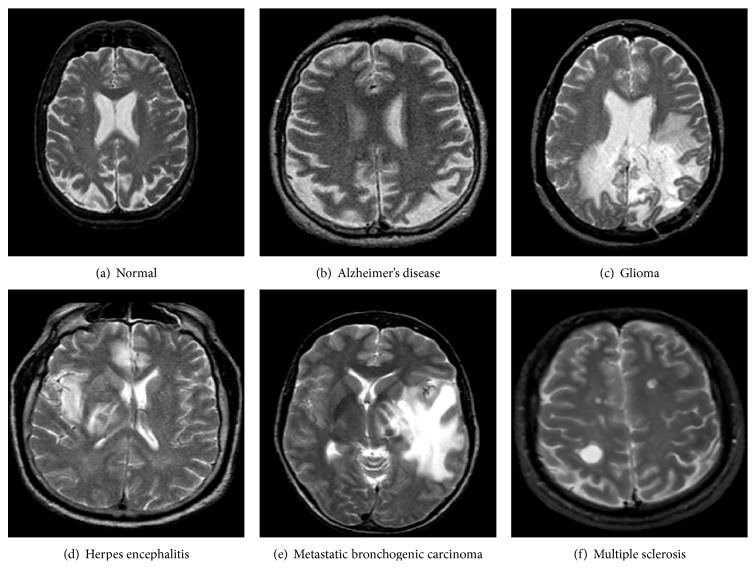
Examples of brain MR images.

**Figure 2:**
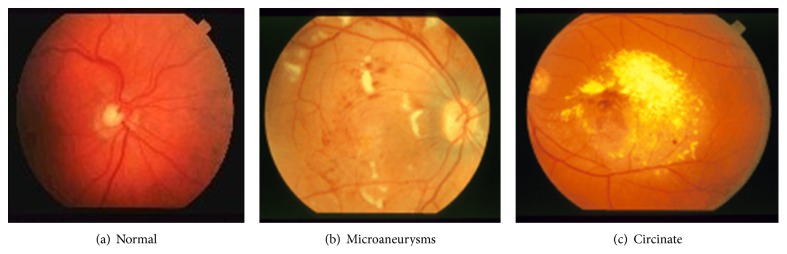
Examples of retina images.

**Figure 3:**
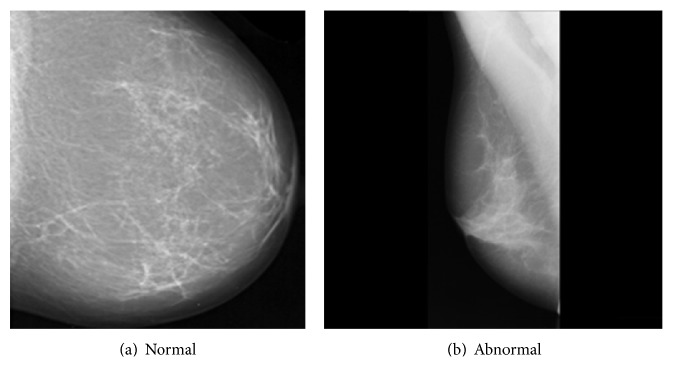
Examples of mammograms.

In a preliminary work [37], the previously mentioned DWT-Gabor hybrid system was successfully applied to mammograms to extract features that allow discriminating normal and cancer images. More specifically, the goal was to detect the presence of malign microcalcifications (specs of calcium in the breast tissue that appear in the mammogram as small bright spots that are scattered or grouped in clusters), whose early detection is important for cancer screening [38, 39]. The results showed the superiority of the approach over simply using the DWT alone. In the present work, we widen our study to retina digital images and brain magnetic resonance images to investigate the effectiveness of the DWT-Gabor approach across application domains with similar image features. Indeed, the images of some pathologies related to brain, retina, and breast present similar contrast features characterized by abrupt changes in image texture with directional properties (see examples in Figures 1, 2, and 3). For instance, breast cancer is characterized by dense concentration of contrast cells in the biological tissue, cancer in brain magnetic resonance images is often characterized by large cells with high contrast, and many forms of retinopathy are characterized by the presence of spots on the retina or covering the macula. As a result, the DWT-Gabor hybrid system we have used in our previous work [37] to detect cancer in mammograms could potentially also be applied to brain magnetic resonance images and retina digital images with similar properties. Next is a brief description of the pathologies that were studied in this work.

Circinate retinopathy is a retinal degeneration characterized by a circle of white spots encircling the macula that causes complete foveal blindness [40]; retinal microaneurysms are due to a swelling of the capillaries caused by a weakening of the vessel wall [41] and are considered to be the earliest sign of diabetic retinopathy, among others. Magnetic resonance imaging (MRI) is a noninvasive imaging modality largely used for brain imaging to detect diseases such as Alzheimer's and multiple sclerosis [6, 8]. Alzheimer's disease is the most frequent cause of age-related dementia and multiple sclerosis is a progressive neurological disorder that can result in various dysfunctions [42]. Additional brain pathologies that can be detected from MR images and that are investigated in this work include glioma, herpes encephalitis, and metastatic bronchogenic carcinoma (Figure 1). All of these are characterized by large cells with high contrast, hence the interest in being able to detect them with the same algorithm.

The contribution of our work can be summarized as follows. First, we propose a relatively simple and fast approach to biomedical image characterization that relies on the directional properties of high-frequency components. The DWT is applied first to extract high-frequency components that characterize abrupt changes in the biological tissue and, then, the Gabor filter is applied to the obtained HH subimage to extract directional features. Second, the statistical features extracted from the hybrid DWT-Gabor transform are processed by an SVM for classification. This statistical binary classifier has proven its efficiency [4–6, 32–35, 37] and ease of tuning in comparison to alternatives such as artificial neural networks. Another desirable feature is its scalability and ability to avoid local minima [36]. Third, contrary to alternatives that focus on ROIs or specific image details, the proposed methodology is of more general reach as three different types of images used for validation show.

The paper is organized as follows.  Section 2 reviews previous works related to the automatic classification of normal versus abnormal images in the context of brain magnetic (MR) resonance imaging, mammograms, and retina digital images.  Section 3 describes our proposed approach for directional features extraction from biomedical images using discrete wavelet transform followed by Gabor filter banks and support vector machines classifier.  Section 4 presents experimental results. Finally Section 5 draws the conclusions and gives future work to be done.

## 2. Related Works

Mammograms, retina, and MR images are the subject of many research efforts on feature extraction and subsequent classification. Next is a summary of some recent works related to DWTs and/or Gabor filters. In the problem of automatic classification of mammograms, the authors in [3] used Gabor filter banks to process images and *k* nearest neighbour (*k*-NN) algorithm as classifier. The obtained classification rate was 80%. In [4] the dual-tree complex wavelet transform (DT-CWT) and support vector machine (SVM) were employed to classify benign and malignant images. The experimental result achieved 88.64% classification accuracy. The authors in [5] employed the contourlet transform and successive enhancement learning (SEL) weighted SVM to obtain 96.6% correct classification rate. The previous studies all used images of size 1024 × 1024 pixels.

In the problem of retina digital image classification, the authors in [1] employed the Belkyns's shift-invariant DWT to classify normal against abnormal retina images of size 700 × 605 pixels. The pathologies of the abnormal images included exudates, large drusen, fine drusen, choroidal neovascularization, central vein and artery occlusion, histoplasmosis, arteriosclerotic retinopathy, hemicentral retinal vein occlusion and more. In order to capture texture directional features, they employed normalized gray level cooccurrence matrices (GLCMs). The obtained classification accuracy with linear discriminant analysis (LDA) was 82.2%. The authors in [2] employed the probabilistic boosting algorithm and morphological scale space analysis and GLCM to extract texture features. The purpose was to classify normal images versus drusen images with various texture complexities. The detection accuracy of normal images varied between 81.3% and 92.2%, and that of abnormal images varied between 71.7% and 85.2% depending on texture complexity (grade of pathology). The authors in [43] used four approaches to extract features from retina digital images of size 300 × 300 pixels to automatically classify glaucoma images. The first set of features is obtained by taking the pixel intensities as input to principal component analysis. The second features are obtained from Gabor texture filter responses. The third set of features is computed from the coefficients of the fast Fourier transform and the fourth set of features is obtained from the histogram of the intensity distribution of the image. Finally, support vector machines were employed for the classification task. The performance of the classifications using one feature set only was 73% with the histogram features, 76% with the fast Fourier transform coefficients, 80% with the Gabor textures, and 83% with the pixel intensities.

Finally, in the problem of brain MRI classification, the authors in [6] used the wavelet coefficients as input to a support vector machine to classify normal and abnormal Alzheimer's disease images of size 256  ×  256 pixels. The classification accuracy was 98% using SVM with a radial basis kernel. More recently, the authors in [42] used voxels to represent each brain MRI of size 512 × 512 pixels. Using cross-validated tests, the obtained correct classification rates of normal and Alzheimer images were 90%, 92%, and 78%, respectively, when using classification by SVM, naïve Bayes classifier, and voting feature intervals (VFIs). Still more recently, the authors in [8] employed the DWT to extract features from brain magnetic resonance images of size 256 × 256 pixels, and then principal component analysis was used to reduce the dimensions of the features space. The abnormal images included glioma, meningioma, Alzheimer's, Alzheimer's plus visual agnosia, Pick's disease, sarcoma, and Huntington's disease. The classification accuracies using backpropagation neural network (BPNN) were 100% using learning and testing sets of 33 images each.

In this work, we are interested in how a DWT-Gabor-based approach for feature extraction may provide better classification results than those reported in the previous works, particularly those based on the DWT alone. The next section provides the details of our methodology.

## 3. Methodology

**Figure 4:**
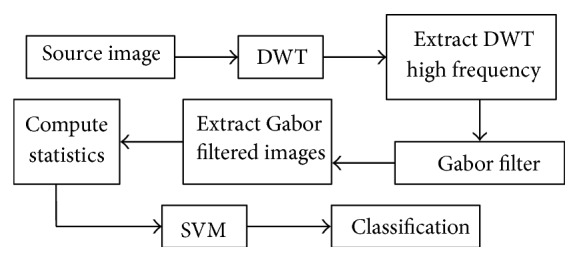
Schematic diagram of the DWT-Gabor approach.

**Figure 5:**
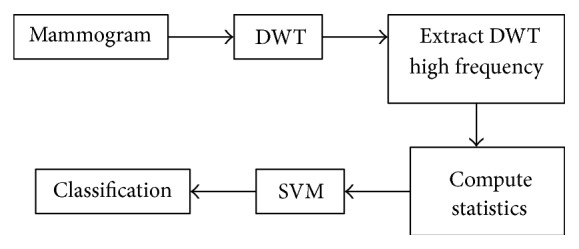
Schematic diagram of the DWT approach.

The overall methodology proceeds as follows. First, the DWT is applied to the biomedical image to obtain its high-frequency image component since it often contains most of the desired information about the biological tissue [39]. Indeed, sudden changes in the texture of the image are typical indicators of the presence of abnormal biological tissue. Second, a bank of Gabor filters with different scales and orientations is applied to the high-frequency image to obtain Gabor-filtered images along different spatial orientations. Third, statistical features are extracted from the Gabor-filtered images. Finally, the SVM is used to classify the resulting feature vector for final diagnosis. The block diagram of the DWT-Gabor system is shown in Figure 4.  Figure 5 summarizes the DWT approach in comparison.

### 3.1. Discrete Wavelet Transform

**Figure 6:**
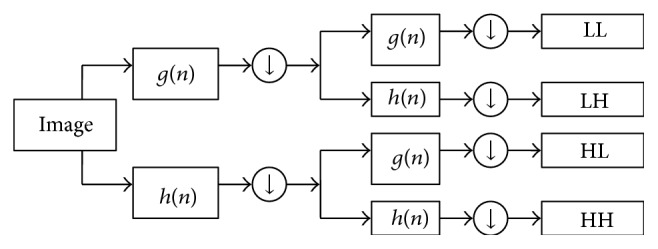
2D-DWT decomposition of an image.

The two-dimensional discrete wavelet transform (2D-DWT) [14–16] performs a subband coding of an image in terms of spectral spatial/frequency components, using an iterative and recursive process.  Figure 6 illustrates the case of two-level decomposition. The image is first represented by LH, HL, and HH subbands that encode the image details in three directions and an LL subband which provides an approximation of it. The obtained detail or approximation images can be decomposed again to obtain second-level detail and approximation images, and the process can be repeated for finer analysis as each iteration doubles the image scale.

The computation of the 2D-DWT proceeds from that of the 1D-DWT, the discrete version of the one-dimensional continuous wavelet transform. The one-dimensional continuous wavelet transform of a signal *x*(*t*) is defined by [7, 8]

(1) $$\begin{array}{c} W_{\psi }\left(a,b\right)=\int_{-\infty }^{+\infty }x\left(t\right)\psi_{a,b}^{*}\left(t\right)dt, \end{array}$$

(2) $$\begin{array}{c} \psi_{a,b}\left(t\right)=\frac{1}{\sqrt{\left|a\right|}}\psi \left(\frac{t-b}{a}\right), \end{array}$$

where *ψ*_*a*,*b*_(·) stands for a given wavelet function and *a* and *b* are the scale and translation parameters, respectively. The 1D-DWT is obtained by sampling *a* and *b* so that (1) becomes that of a sequence. In dyadic sampling, *a* and *b* are, respectively, a power of 2 and multiples thereof, and the sequence elements (wavelet coefficients) are given by

(3a) $$\begin{array}{c} c_{jk}=W_{\psi }\left(2^{-j},2^{-j}k\right), \end{array}$$

where *j* represents the discrete scale factor and *k* the discrete translation factor. In other words, *a* and *b* in (1) are replaced by 2^*j*^ and 2^*j*^ *k*, respectively.

The one-dimensional wavelet decomposition is extended to an image by applying it to the row variable first and then to the column variable of the obtained result [44]. At each step, two subimages are created with half the number of pixels of the row or column that was processed. In the end, an *M* × *N* image is decomposed into 4 subimages, each with *M*/2 × *N*/2 resolution and preserved scale. However, (1) has only theoretical merit due to the infinite ranges of *a* and *b*. For a practical implementation, the fact that (1) is essentially a measure of correlation between a signal and various wavelets derived from a mother is exploited, and the DWT decomposition is turned into a filtering operation with a sequence of high-pass and low-pass filters [45]. Following the notation in [7, 8], the discrete form of (1) can then be written as

(4) $$\begin{array}{c} ca_{j,k}\left[x\left(t\right)\right]=\text{DS}\left[\sum x\left(t\right)g_{j}^{*}\left(t-2^{j}k\right)\right],\\ cd_{j,k}\left[x\left(t\right)\right]=\text{DS}\left[\sum x\left(t\right)h_{j}^{*}\left(t-2^{j}k\right)\right], \end{array}$$

where coefficients *ca*_*j*,*k*_ and *cd*_*j*,*k*_ specify approximation and details components provided by the *g*(*n*) low-pass and *h*(*n*) high-pass impulse responses, respectively, and the DS operator performs downsampling by a factor of 2. The one-dimensional wavelet decomposition is extended to two-dimensional objects by using row and column decompositions as shown in Figure 6. In our work, the most frequently used wavelet (Daubechies-4) [25] is considered to extract the HH image component.

### 3.2. Gabor Filter

The two-dimensional (2D) Gabor filter decomposes an image into components corresponding to different scales and orientations [22], thus capturing visual properties such as spatial localization, orientation selectivity, and spatial frequency. The 2D Gabor filter consists of a complex exponential centered at a given frequency and modulated by a Gaussian envelope. Because of the complex exponential, the filter has both real and imaginary parts. The general form of the real part is defined as follows:

(5) G(x,y,σx,σy,f,θ)=exp[−12((x′σx)2+(y′σy)2)]×cos(2πfx′),

where

(6) $$\begin{array}{c} x^{\prime }=x\cos\left(\theta \right)+y\sin\left(\theta \right),\\ y^{\prime }=y\cos\left(\theta \right)-x\sin\left(\theta \right) \end{array}$$

and where *σ*_*x*_ and *σ*_*y*_ are the standard deviations of the Gaussian envelope along the *x* and *y* axes. The parameters *f* and *θ* are, respectively, the central frequency and the rotation of the Gabor filter. To obtain the Gabor-filtered image *f*(*x*, *y*) of a given image *i*(*x*, *y*) the 2D convolution operation (∗) is performed:

(7) $$\begin{array}{c} f\left(x,y\right)=G\left(x,y,\sigma_{x},\sigma_{y},f,\theta \right)*i\left(x,y\right). \end{array}$$

The selection of parameters *σ*_*x*_, *σ*_*y*_, *f*, and *θ* plays an important role in the filter's operation. However, no formal technique exists for choosing them and experience-guided intuition, trial and error, or heuristic search must be used. For retina digital images and brain MR images, *σ*_*x*_ and *σ*_*y*_ were arbitrarily set to unity. In the case of mammograms, *σ*_*x*_ and *σ*_*y*_ were set to the values used in [46], which were determined empirically. Consequently, we used *σ*_*x*_ = *τ*/2.35, where *τ* is the full width at half-maximum of the Gaussian and *σ*_*y*_ = 8*σ*_*x*_. No values of *σ*_*x*_ and *σ*_*y*_ other than the previous ones were tried since optimality was not the primary concern of this work and we obtained satisfactory results with these values.

Four orientations, *θ* = 0, *π*/4, *π*/2, and 3*π*/4, were used as in [22, 33]. These values seemed reasonable as a first try since they covered both image axis directions and the forward and backward diagonals. Finally, the central frequency *f* was set to 2, 2.5, and 3. Given that the Gabor filter is modulated by the cosine of *f*, large values of *f* lead to a compressed cosine and, consequently, the filter output is more likely to show fast or frequent changes in biological tissue texture. This in turn would help verify our hypothesis that abnormal images are characterized by sudden and frequent variations in image texture. In the end, the application of the Gabor filter bank to the HH image component obtained with the 2D-DWT leads to twelve Gabor-filtered HH images components, for each choice of *f* and *θ*.

### 3.3. Feature Extraction

Statistical measures are employed to extract features from both the DWT HH subband image and the real Gabor-filtered HH image components. More precisely, the entropy (*E*) and uniformity (*U*) of the coefficients of each one are computed. Entropy and uniformity were selected as features because previous works on mammograms have shown that uniformity is correlated with suspicious malignancy [47] and that entropy can successfully characterize breast biological tissue [48]. In this study, the entropy and uniformity statistics are hypothesized to also characterize retina and brain MR images with similar contrast information (i.e., abrupt and/or frequent variations in texture). Entropy (*E*) and uniformity (*U*) are defined by [49]

(8) $$\begin{array}{c} E=-\sum p\left(z\right)\times \log\left(p\left(z\right)\right),\\ U=\sum p^{2}\left(z\right), \end{array}$$

where *z* is a random variable that represents a coefficient in the Gabor filtered image and *p*(*z*) is its probability of occurrence as estimated by its relative frequency.

To investigate the performance of the previous approaches, the image features were extracted from HH at both level-one DWT decomposition (HH1) and level-two DWT decomposition (HH2), with and without filtering by a Gabor filter bank. We also applied the Gabor filter directly to the original image without the DWT for comparison purpose. For each DWT HH subband image, the feature vector is given by

(9) $$\begin{array}{c} V_{\text{DWT},a}=\left[E_{a},U_{a}\right], \end{array}$$

where *a* is the level of wavelet analysis (decomposition). Similarly, for each of the twelve outputs generated by the Gabor filter bank (4 angles × 3 central frequencies), the entropy and uniformity are computed and a twenty-four component feature vector is formed to represent the initial image. We thus have

(10) $$\begin{array}{c} V_{\text{Gabor},a}=\left[E_{1,a},E_{2,a},\ldots ,E_{12,a},U_{1,a},U_{2,a},\ldots ,U_{12,a}\right]. \end{array}$$

Either feature vector is subsequently fed to the SVM to classify normal versus pathological images.

### 3.4. The Support Vector Machine Classifier

Introduced by Vapnik [36], the support vector machine (SVM) classifier is based on statistical learning theory. It implements the principle of structural risk minimization and has excellent generalization ability as a result, even when the data sample is small. Moreover, SVM can tolerate high-dimensional and/or incomplete data [50]. It has been used with great success in various applications, including speech emotion recognition [51], card-sharing traffic detection [52], fault diagnosis [53], cardiac decision making [54], Parkinson's disease diagnosis [55], and Alzheimer's disease detection [56].

The support vector machine performs classification tasks by constructing an optimal separating hyperplane that maximizes the margin between the two nearest data points belonging to two separate classes. Given a training set {(*x*_*i*_, *y*_*i*_), *i* = 1,2,…, *m*}, where the input *x*_*i*_ ∈ *R* ^*d*^ and class labels *y*_*i*_ ∈ {+1, −1}, the separation hyperplane for a linearly separable binary classification problem is given by

(11) $$\begin{array}{c} f\left(x\right)=\langle w\cdot x\rangle +b, \end{array}$$

where *w* is a weight vector and *b* is a bias. The optimal separation hyperplane is found by solving the following optimization problem:

(12)  minimizew,b,ξ  12〈w·w〉+C∑i=1mξi Subject  to: yi(〈w·xi〉+b)+ξi−1≥0, ξi≥0,

where *C* is a penalty parameter that controls the tradeoff between the complexity of the decision function and the number of misclassified training examples and *ξ* is a positive slack variable. The previous optimization model can be solved by introducing Lagrange multipliers and using the Karush-Kuhn-Tucker theorem of optimization to obtain the solution as

(13) $$\begin{array}{c} w=\sum_{i=1}^{m}\alpha_{i}y_{i}x_{i}. \end{array}$$

The *x*_*i*_ values corresponding to positive Lagrange multipliers *α*_*i*_ are called support vectors which define the decision boundary. The *x*_*i*_ values corresponding to zero *α*_*i*_ are irrelevant. Once the optimal solution *α*_i_^*^ is found, the optimal hyperplane parameters *w* ^*^ and *b* ^*^ are determined. Then, the discriminant function of the SVM for a linearly separable binary classification problem is [32]

(14) $$\begin{array}{c} g\left(x\right)=\mathrm{sign}\left(\sum_{i=1}^{m}y_{i}\alpha_{i}^{*}\langle x_{i}\cdot x\rangle +b^{*}\right). \end{array}$$

In the nonlinearly separable case, the SVM classifier nonlinearly maps the training points to a high-dimensional feature space using a kernel function Φ, where linear separation can be possible. The scalar product 〈Φ(*x*_*i*_) · Φ(*x*_*j*_)〉 is computed by Mercer kernel function *K* as *K*(*x*_*i*_, *x*_*j*_) = 〈Φ(*x*_*i*_) · Φ(*x*_*j*_)〉. Then, the nonlinear SVM classifier has the following form:

(15) $$\begin{array}{c} g\left(x\right)=\mathrm{sign}\left(\sum_{i=1}^{m}y_{i}\alpha_{i}^{*}K\langle x,x_{i}\rangle +b^{*}\right). \end{array}$$

In this study, a polynomial kernel of degree 2 was used for the SVM. As a global kernel, it allows data points that are far away from each other to also have an influence on the kernel values. The general polynomial kernel is given by

(16) $$\begin{array}{c} K\left(x,x_{i}\right)={\left(\left(x_{i}\cdot x\right)+1\right)}^{d}, \end{array}$$

where *d* is the degree of the polynomial to be used.

## 4. Experimental Results

**Figure 7:**
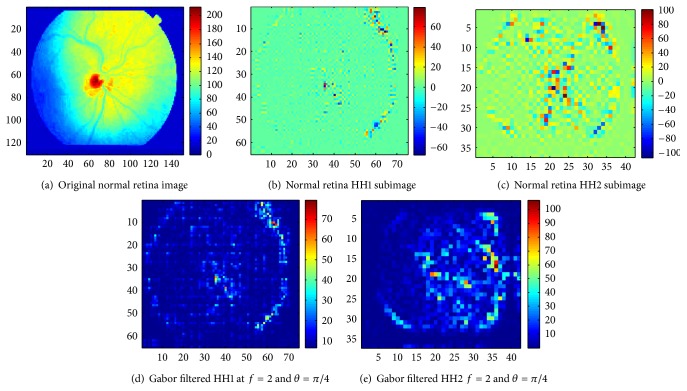
Analysis of a normal retina.

**Figure 8:**
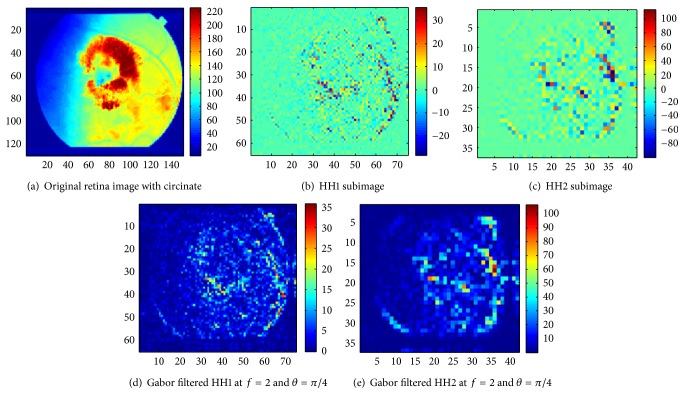
Analysis of a retina with circinate.

As mentioned previously, mammograms and retina and brain MR images corresponding to given pathologies are considered in this work, and the aim is to classify normal versus abnormal images for each image category. To do so, one hundred digital mammograms (171 × 364 pixels) consisting of fifty normal images and fifty cancer images were taken from The Digital Database for Screening Mammography (DDSM) [57]. For retina, a set of 69 color images (150 × 130 pixels) from the STARE [58] database were employed including 23 normal images, 24 with microaneurysms, and 22 with circinate. Finally, a collection of 56 axial, T2-weighted, and MR brain images (256 × 256 pixels) were taken from the AANLIB database [59] of the Harvard Medical School. They consisted of 7 normal images, 9 with Alzheimer's disease, 13 with glioma, 8 with Herpes encephalitis, 8 with metastatic bronchogenic carcinoma, and 14 with multiple sclerosis. It is unfortunate that the number of images was not constant across pathologies, but we had no control over this and used what was available, with tenfold cross-validation or leave-one-out cross-validation of the results depending on sample size. All experiments were based on a binary classification approach of normal versus abnormal images. Many kinds of biomedical images could be considered for our experiments; we focused on mammograms, retina, and brain magnetic resonance images mainly because of public availability. An example of the processing of a normal retina and a retina with circinate is illustrated in Figures 7 and 8, respectively.

For each image type, the average and standard deviation of the correct classification rate (CCR), sensitivity, and specificity were computed to evaluate the performance feature extraction technique when used in conjunction with the SVM classifier. The three performance measures are defined by

(17) $$\begin{array}{c} \text{CCR}=\frac{\text{Classified}\;\text{Samples}}{\text{Total}\;\text{Number}\;\text{of}\;\text{Samples}},\\ \text{Sensitivity}=\frac{\text{Correctly}\;\text{Classified}\;\text{Positive}\;\text{Samples}}{\text{True}\;\text{Positive}\;\text{Samples}},\\ \text{Specificity}=\frac{\text{Correctly}\;\text{Classified}\;\text{Negative}\;\text{Samples}}{\text{True}\;\text{Negative}\;\text{Samples}}, \end{array}$$

where positive samples and negative samples are, respectively, abnormal and normal images.

Finally, all experiments were performed with tenfold cross-validation, except those for MR images which used leave-one-out cross-validation due to the small sample size of each brain image category.

Table 1 shows the obtained average results for the three types of images that were investigated. The performance of the SVM classifier improved for all types of images and all levels of HH decomposition by the DWT. At level one, the average correct classification rate increased by, respectively, 0.69, 25.31, and 9.56 percentage points for mammograms, retina, and brain magnetic resonance images when using the DWT-Gabor approach. At level-two decomposition, the improvement was, respectively, 1.96, 9.02, and 5.42 percentage points.

Tables 2 and 3 provide the average results for classifier sensitivity and specificity. At level-one DWT decomposition, the DWT-Gabor approach improved classification specificity for mammograms and retina images to make it reach 100%, while it improved it by 78.03 percentage points for brain MR images. At level-two DWT decomposition, the improvement was 3.24 percentage points for mammograms, 97.68 percentage points for retina images (100% specificity), and 97.24 percentage points for MR images. Regarding sensitivity, the results were mixed.

At level-one DWT decomposition, the values were about the same for the DWT-Gabor and DWT-only approaches for mammograms and retina images, with, respectively, −0.81 and 0 percentage points differences, but there was a degradation of −11.33 and −46.16 percentage points for brain MR images at level one and level two of decomposition, respectively.

Following the same cross-validation protocol, we also conducted classification experiments with features extracted from a Gabor filtered image of the original biomedical image. The purpose was to check whether Gabor-based features alone help characterize images better than DWT or DWT-Gabor-based features. The results are given in Table 4. The obtained correct classification rate of mammograms, retina, and brain magnetic resonance images is, respectively, 68.03% (±0.01), 50.00% (±0.00), and 86.61% (±0.03). The average results for classifier specificity and sensitivity for all images are 100% and 0%. This finding indicates that Gabor-based features are suitable to detect pathological images, but fails to detect normal images. In sum, the results show that Gabor-based features do not perform better than DWT and DWT-Gabor-based features. These findings confirm the superiority of combining the DWT and Gabor filter banks for feature extraction.

Based on the previous results, it appears that the DWT-Gabor approach for feature extraction is effective for detecting the abrupt changes in biological tissue that characterize the pathological patterns that were investigated and it yields better classification accuracy and specificity than the DWT-only approach. It also offers equal of better sensitivity, except for brain MRIs. For brain MRIs, the obtained specificity and sensitivity results with the DWT-Gabor approach show improved true negative detection, but lower true positive performance. Finally, the obtained results reveal also that level-one DWT decomposition is preferable to level-two decomposition.

Finally, Table 5 compares the results obtained with the DWT-Gabor approach to other work that we surveyed. In many cases, the DWT-Gabor method yields higher classification rates, particularly for mammograms and retina. For the problem of brain MRI classification, our obtained performance is better than the results of [38], but less than what is reported in [6, 8]. However, these comparisons should be viewed with caution as not all the results stem from a common image database and the different authors use different sample and image sizes. Moreover, many authors use no cross-validation and simply perform a single arbitrary split of their data into training and test sets to obtain their accuracy results. Obviously, one cannot generalize or draw definite conclusion from such efforts, and comparisons between works cannot be made other than in general terms. In this respect, it can only be concluded from our results that the DWT-Gabor for feature extraction is effective for obtaining high image classification accuracy by an SVM and that it may outperform other feature extraction and classification techniques reported in the literature, at least those based on DWT-only image decomposition. Unfortunately, a more definite conclusion is impossible without gaining access to the image databases used by the other authors.

## 5. Computational Complexity

Finally, the computational complexity of the DWT, Gabor, contourlet, and curvelet for an *N* × *N* image is, respectively, *O*(*N*), *O*(*N* ^2^ × *M* ^2^) with *M* being the width of Gabor (Gaussian) mask filter, *O*(*N* ^2^), and *O*(*N* ^2^log(*N*)). As a result, the computational complexity of the combination of the DWT and Gabor filter is *O*(*N*) + *O*(*N* ^2^ × *M* ^2^). In terms of features extraction processing time, the average time required to process a brain, a mammogram, and a retina image with the DWT approach (DWT-Gabor) was, respectively, 0.19 (0.31), 0.17 (0.32), and 0.15 seconds (0.35) using Matlab R2009a on a 1.5 GHz Core2 Duo processor.

## 6. Conclusion

We proposed a supervised system for biomedical images classification that uses statistical features obtained from the combination of the discrete wavelet transform and Gabor filter to classify normal images versus cancer images, using support vector machines as classifiers. Our experimental results show that such a hybrid processing model achieves higher accuracy in comparison to using DWT or Gabor filter banks alone. Therefore, the proposed image processing and features extraction approach seem to be very promising for the detection of certain pathologies in biomedical images.

For future works, it is recommended to consider a larger set of features and a selection process to identify the most discriminant ones. In addition, the Gabor parameters will be adjusted for each type of image separately to improve the accuracy. Furthermore, the DWT-Gabor will be directly compared to the dual-tree complex wavelet, curvelet, and contourlet using the same databases and images in order to draw general conclusions. Also, multilabels classifications will be considered in future works to investigate the discriminative power of our approach for each type of pathology. Finally, more experiments on the effect of kernel choice and its parameter on classification accuracy will be investigated.

## Figures and Tables

**Table 1:** Average SVM classification accuracy as a function of feature extraction method and level of DWT decomposition∗.

	DWT	DWT-Gabor	DWT	DWT-Gabor
Decomposition level	One	One	Two	Two
Mammograms	95.98% (±0.04)	96.67% (±0.05)	89.13% (±0.01)	91.09% (±0.05)
Retina	74.69% (±0.05)	100%	90.98% (±0.03)	100%
Brain MRI	87.80% (±0.00)	97.36% (±0.02)	85.76% (±0.00)	91.18% (±0.04)

^*^Tenfold cross-validation used for mammograms and retina images, leave-one-out used for brain MRIs.

**Table 2:** SVM classification specificity as a function of feature extraction method and level of DWT decomposition∗.

	DWT	DWT-Gabor	DWT	DWT-Gabor
Decomposition level	One	One	Two	Two
Mammograms	97.81% (±0.02)	100%	88.85% (±0.05)	92.09% (±0.06)
Retina	6.81% (±0.04)	100%	2.32% (±0.10)	100%
Brain MRI	21.55% (±0.01)	99.58% (±0.01)	0%	97.24% (±0.01)

^*^Tenfold cross-validation used for mammograms and retina images, leave-one-out used for brain MRIs.

**Table 3:** SVM classification sensitivity as a function of feature extraction method and level of DWT decomposition∗.

	DWT	DWT-Gabor	DWT	DWT-Gabor
Decomposition level	One	One	Two	Two
Mammograms	94.14% (±0.06)	93.33% (±0.06)	90.29% (±0.039)	89.78% (±0.04)
Retina	100%	100%	100%	100%
Brain MRI	93.84% (±0.00)	82.51% (±0.16)	100%	53.84% (±0.23)

^*^Tenfold cross-validation used for mammograms and retina images, leave-one-out used for brain MRIs.

**Table 4:** SVM classification performance measures obtained with Gabor-based features.

	Accuracy	Specificity	Sensitivity
Mammograms	68.03% (±0.01)	100%	0%
Retina	50.00% (±0.00)	100%	0%
Brain MRI	86.61% (±0.03)	100%	0%

**Table 5:** Comparison with the literature.

	Features	Classifier	Accuracy∗
Mammograms			
[3]	Gabor	*k*-NN	80%
[4]	DT-CWT	SVM	88.64%
[5]	Contourlet	SVM	96.6%
Our approach	DWT-Gabor	SVM	96.67% (±0.05)
Retina			
[1]	DWT + GLCM	LDA	82.2%
[2]	Morphological + GLCM	Probabilistic boosting algorithm	81.3%–92.2% 71.7%–85.2%
[39]	Gabor	SVM	83%
Our approach	DWT-Gabor	SVM	100%
Brain			
[6]	DWT	SVM	98%
[8]	DWT + PCA	BPNN	100%
		SVM	90%
[38]	Voxels	Bayes	92%
		VFI	78%
Our approach	DWT-Gabor	SVM	97.36% (±0.02)

^*^Correct classification rate.
